# Efficacy of combination of endo-xylanase and xylan-debranching enzymes in improving cereal bran utilization in piglet diet

**DOI:** 10.5713/ab.21.0534

**Published:** 2022-06-30

**Authors:** Weiwei Wang, Dawen Zheng, Zhenzhen Zhang, Hui Ye, Qingyun Cao, Changming Zhang, Zemin Dong, Dingyuan Feng, Jianjun Zuo

**Affiliations:** 1 Guangdong Provincial Key Laboratory of Animal Nutrition Control, College of Animal Science, South China Agricultural University, Guangzhou 510642, China; 2 AsiaPac Bio-Technology Co. Ltd, Dongguan 523808, China

**Keywords:** Arabinoxylan, Bran, Debranching Enzyme, Intestine, Piglet, Xylanase

## Abstract

**Objective:**

This study was aimed to explore the efficacy of combination of endo-xylanase (Xyn) and xylan-debranching enzymes (arabinofuranosidase, Afd and feruloyl esterase, FE) in improving utilization of bran in piglet diet.

**Methods:**

*In vitro* experiments were firstly conducted to examine the enzymological properties of Xyn, Afd, and FE, concurrent with their effect on degradation of arabinoxylan (Abx) in bran. *In vivo* experiment was then implemented by allocating two hundred and seventy 35-d-old postweaning piglets into 3 groups (6 replicates/group), which received bran-containing diet supplemented with Xyn (1,600 U/kg) or its combination with Afd (0.8 U/kg) and FE (4 U/kg) or without enzyme.

**Results:**

Both Xyn, Afd, and FE are relatively stable against the changes in temperature and pH value. Combining Xyn with Afd and FE had a superiority (p<0.05) over Xyn alone and its combination with Afd or FE in promoting (p<0.05) degradation of Abx in different brans. Combined treatment with Xyn, Afd, and FE was more beneficial than Xyn alone to induce increasing trends (p<0.10) of average daily gain, final body weight and feed efficiency of piglets fed bran-containing diet. Moreover, combination of Xyn, Afd, and FE showed advantages (p<0.05) over Xyn alone in causing reductions (p<0.05) in diarrhea rate and cecal pH value, concurrent with increases (p<0.05) in cecal and colonic acetic acid and total volatile fatty acid concentrations, as well as cecal butyric acid concentration of piglets fed bran-containing diet.

**Conclusion:**

Combining Xyn with Afd and FE was more beneficial than Xyn alone in promoting degradation of Abx in bran, along with growth performance and intestinal volatile fatty acid profile of piglets received bran-containing diet. Thereby, combination of Xyn, Afd, and FE had a superior efficacy relative to Xyn alone in improving application of cereal bran in piglet diet.

## INTRODUCTION

Despite the usage of cereal bran as a feedstuff, the existence of high level of arabinoxylan (Abx) may limit its application in animal diets [1], because Abx is resistant to digestion by endogenous digestive enzymes. More importantly, Abx can cause viscous digesta with subsequent intestinal disorders such as reduced digestibility of nutrients, increased pathogen load, gut leakage, and inflammation [2,3]. Thereby, Abx is considered as a critical anti-nutritional factor for monogastric animals especially for those at young age (e.g., piglets) [2]. These raise a necessity to explore strategies to efficiently degrade Abx in cereal bran that may benefit its application in animal diets.

It is known that Abx represents a typical member of non-starch polysaccharides in multiple plant ingredients, comprising a linear backbone of β-D-(1,4)-xylopyranosyl units onto which L-arabinose units are linked as the side chains [4]. Generally, arabinose residues that are attached to the C-2, C-3, or both positions of Abx backbone can be further substituted in the O-5 position by ferulic acid ester groups [4]. Because of the complex molecular structure, the efficient degradation of dietary Abx may demand a combination of depolymerizing enzymes such as endo-xylanase (Xyn) with debranching enzymes such as arabinofuranosidase (Afd), and feruloyl esterase (FE) [5,6]. Thereinto, Xyn stochastically cleaves the β-1,4-glycosidic bonds within Abx backbone and produces smaller polysaccharides or oligosaccharides [5], which can be used as prebiotics to improve intestinal health by targeting host gut microbiota [2]. However, the presence of side chains enhances the degradation resistance of Abx backbone via impeding recognition of cleavage sites in the backbone by Xyn [6,7]. This effect may be overwhelmed by the action of debranching enzymes, among which Afd removes the arabinose residues to expose more cleavage sites and provide a convenience for Xyn action [6,7], while FE breaks ferulic acid ester bonds cross-linked to arabinose residues to liberate ferulic acid [8]. These actions aid in simplifying the molecular structure of Abx and enhancing Xyn accessibility to produce reducing sugars [8–10]. Accordingly, there can be an efficient degradation of Abx in plant-sourced ingredients under the synergy among Xyn, Afd, and FE, which may in turn diminish the viscosity of intestinal content, thus promoting a sufficient contact between digesta and digestive enzymes with a resultant benefit on nutrient digestion [2,3]. In addition, combination of Xyn, Afd, and FE may prompt plant cell wall fragmentation by efficiently degrading Abx and cleaving the ferulic acid ester bonds linking feruloyl residues in Abx with other cell wall components (e.g., cellulose), presumptively favoring the access of digestive enzymes to the cell wall or intracellular components [8–10]. Thereby, combination of Xyn, Afd, and FE might be superior to Xyn alone for enhancing digestion of cereal bran with subsequent promotions of growth and health of animals ingesting a bran-containing diet. Although there were numerous studies revealing the positive effects of Xyn and its combination with Afd on chicken growth and health performance [11,12], there were few studies regarding the combined application of Xyn, Afd, and FE in pig diets.

Comprehensively, we raised the hypothesis that combination of Xyn, Afd, and FE was more favorable to promote degradation of Abx with a destruction of cell wall of cereal bran, possibly ameliorating the application effect of cereal bran. To test this hypothesis, the current study was conducted to investigate the effects of a combination of Xyn, Afd, and FE on degradation of Abx-riched cereal bran as well as growth and health of piglets received bran-containing diet.

## MATERIALS AND METHODS

### *In vitro* experiments

#### Enzymatic characteristics assay

The activity of Xyn (EC 3.2.1.8; AsiaPac, Dongguan, China) was determined in the reaction mixture containing 200 μL properly diluted enzyme and 1.8 mL of 10 mg/mL xylan (Sigma, St. Louis, MO, USA) in 0.05 M citric-Na_2_HPO_4_ (pH 5.0). After incubation at 40°C for 10 min, the enzymatic activity was terminated, and the reducing sugar was quantified using the dinitrosalicylic acid (DNS) method [13]. The activity of Afd (EC 3.2.1.55; AsiaPac, China) was determined by using *p*-nitrophenyl α-L-arabinofuranoside (Sigma, USA) as the substrate. The reaction mixture consisted of 25 μL properly diluted enzyme and 175 μL of 2 mM substrate in 0.05 M citric-Na_2_HPO_4_ (pH 5.5). After incubation at 40°C for 15 min, the enzymatic reaction was terminated by adding 200 μL of 2 M Na_2_CO_3_. The concentration of p-nitrophenol was measured by determining the absorbance at a wavelength of 410 nm. For FE (EC 3.1.1.73; AsiaPac, China) activity assay, methyl ferulate (Alfa Aesar, Beijing, China) was used as the substrate [13]. The reaction mixture consisted of 10 μL properly diluted enzyme and 190 μL of 0.1 M substrate in 0.05 M citric-Na_2_HPO_4_ (pH 5.5). After incubation at 40°C for 15 min, 100 μL acetonitrile was added to terminate the enzymatic reaction. Ferulic acid was quantified using liquid chromatography (Shimadzu SIL-20A, Kyoto, Japan). One unit of Xyn, Afd, and FE was defined as the amount of enzyme required to generate 1 μmol of product equivalent per min from the corresponding substrates under the standard assay conditions. Each reaction was performed in triplicate.

The influence of pH on enzyme activities was detected by measuring the relative activities of Xyn, Afd, and FE in 0.05 M citric-Na_2_HPO_4_ buffer (pH value ranging from 3.0 to 8.0) and glycine-NaOH (pH value ranging from 9.0 to 10.0) according to the method described above. Enzyme stability against pH was detected by determining the residual activities of these enzymes after incubation at pH 3.0 for 30 min. The effect of temperature on enzyme activities was determined by measuring the relative activities of Xyn, Afd, and FE at different temperatures (30°C to 90°C) under the standard assay conditions described above. Thermostability of these enzymes was evaluated by determining the residual activities of these enzymes after incubation at 85°C for 3 min.

#### Evaluation of effect of Xyn combined with Xyn or Afd on degradation of Abx in destarched wheat bran

The preparation of destarched wheat bran was based on a previous study [14]. Tubes with destarched wheat bran (0.5 g per tube) were divided into six groups (n = 5): control, Xyn (3 U/g), Xyn + 0.001 U/g of Afd, Xyn + 0.003 U/g of Afd, Xyn + 0.006 U/g of Afd, and Xyn + 0.03 U/g of Afd. Meanwhile, tubes with destarched wheat bran (0.5 g per tube) were divided into another six groups (n = 5): control, Xyn (3 U/g), Xyn + 0.001 U/g of FE, Xyn + 0.003 U/g of FE, Xyn + 0.006 U/g of FE, and Xyn + 0.03 U/g of FE. Each treatment group was then added with phosphate buffer solution to obtain 10 mL reaction system, followed by incubation in a constant temperature shaker (40°C; 120 rpm/min) for 4 h. After centrifugation at 2,500 g for 3 min, the supernatant was collected for quantifying the releasing of reducing sugar (RRS) that represents Abx degradation [13].

#### Determination of synergy among Xyn, Afd, and FE on degradation of Abx in different brans

Wheat bran and oat bran (Xingye Biotech, Dongguan, China) were enzymolyzed and the resulting zymolytes were used to extract the soluble fiber (SF) and insoluble fiber (IF) according to a previous study [15]. For evaluation of the synergy among Xyn, Afd, and FE on the RRS from different Abx sources (SF and IF from wheat bran and oat bran), tubes with 0.5 g of each kind of fiber were randomly allocated into four groups (n = 5): Xyn, Xyn+Afd, Xyn+FE, and Xyn+Afd+FE, which received 3 U/g Xyn, 3 U/g Xyn + 0.001 U/g Afd, 3 U/g Xyn + 0.006 U/g FE, and 3 U/g Xyn + 0.001 U/g Afd + 0.006 U/g FE, respectively (Note: the dosages of Xyn, Afd, and FE were selected according to the results of Tables 2 and 3). Each treatment group was then added with phosphate buffer solution to obtain 10 mL reaction system, the supernatant was separated for quantifying the RRS using DNS method [13].

The value of synergy degree between multiple enzymes was calculated as the ratio of the sum of the activities (generation amount of hydrolysate) of all enzymes to the activity of one of these enzymes [16]. If the ratio is less than 1.0, it reveals a negative synergy between enzymes, while the ratio exceeding 1.0 suggests a positive synergy between enzymes [16].

#### Evaluation of effect of combination of Xyn, Afd, and FE on in vitro digestion of bran-containing diet

Referring to a previous study in pigs [17], the gastric digestion juice of piglets was simulated by preparation of pepsin (737.5 U/mL: Sigma, USA) with hydrochloric acid (pH 2.0). The small intestinal digestion juice was simulated by preparing a mixture of amylase (221.4 U/mL; Sigma, USA), trypsin (69.1 U/mL; Amresco, Solon, OH, USA) and chymotrypsin (8.7 U/mL; Amresco, USA) with deionized water. The large intestinal digestion juice was simulated by preparation of cellulase (0.4 U/mL; Sigma, USA) with deionized water.

The composition and nutrient levels of a bran-containing basal diet of piglets based on the NRC requirement of swine [18] are shown in Table 1. The simulated digestion system-II simulated digestion system (Shenhua Biotech, Shenzhen, China) was employed in simulating digestion of the above diet [17]. In brief, diet (2 g) was put into digestive tube sleeved with the MD34-14 dialysis bag (Viskase, Lombard, IL, USA), followed by digestion with 20 mL of simulated gastric juice together with one of three treatments (n = 5): control (no enzymes), 3 U/g Xyn, and 3 U/g Xyn + 0.001 U/g Afd + 0.006 U/g FE at 39°C for 4 h in the digestion system. Immediately after simulated gastric digestion, the diet was treated by 2.2 mL of simulated small intestinal juice at 39°C for 16 h and simulated large intestinal juice at 39°C for 3.5 h. Following these processes, the undegraded chyme was washed repeatedly with deionized water. Thereafter, the chyme residue in the digestive tube was transferred into a preweighted glass petri dish and dried overnight at 65°C and then dried at 105°C for 5 h to constant weight, which was then cooled to determine the digestibility of nutrients (dry matter, crude protein, crude ash, and gross energy) according to the method of Pan et al [17].

### *In vivo* experiment

*In vivo* experiment was conducted to explore the effects of combination of Xyn, Afd, and FE on growth performance and intestinal health of piglets fed bran-containing diet.

#### Animals and experimental design

The experimental animal protocols for this study were approved by the Animal Care and Use Committee of South China Agricultural University (SCAU20191127). Duroc×(Landrace×Yorkshire) crossbred weaned piglets at 35 d of age were raised in stainless steel cages and maintained in an environmentally controlled room (25°C). After acclimation to the environment and basal diet for 1 week, based on the initial body weight (9.70±0.50 kg), sex and source litter, a total of 270 piglets were allocated into three treatment groups with six replicates (three replicates for each male and female) per group and fifteen piglets per replicate in a randomized complete block design. The groups in the experimental design consisted of control group (received bran-containing diet with no enzymes), Xyn group (received bran-containing diet added with 1,600 U/kg Xyn), and Xyn+Afd+FE group (received bran-containing diet added with 1,600 U/kg Xyn, 0.8 U/kg Afd, and 4 U/kg FE). These enzymes were provided by AsiaPac Co. Ltd. (China) and the enzyme dosages were determined based on several preliminary experiments in pigs. The composition and nutrient levels of bran-containing diet are shown in Table 1. The experiment lasted for 21 d. Piglets had free access to water and feed.

#### Growth performance

Feed consumption and final body weight (FBW) of each replicate were recorded at 21 d of the experiment for calculating average daily gain (ADG), average daily feed intake (ADFI), and gain to feed ratio (G/F). Meanwhile, individual pigs were examined for diarrhea two times per day during the experimental period to calculate the diarrhea rate, which was evaluated by fecal consistency scoring using a four-grade system [19]. Diarrhea occurrence was defined as maintaining a score of 3 for two days or a score of 4 for one day. The diarrhea rate was calculated according to the following formula: diarrhea rate (%) = the number of diarrhea pigs × diarrhea days/(the total number of pigs × experiment days).

#### Intestinal health

At 21 d of the experiment, one piglet per replicate was randomly selected and slaughtered by severing the jugular vein, followed by separation of the intestine. Immediately after cutting open the cecum and colon, the pH value of digesta in cecum and colon was measured with a DELTA320 pH meter (Mettler Toledo, Zurich, Switzerland). The digesta of cecum and colon were then collected for measuring volatile fatty acids (VFA) concentrations using gas chromatography GC-17A (Shimadzu, Japan) with a flame ionization detector fitted with a Nukol FFAP capillary column (30 m ×0.32 mm×0.25 μm; Supelco, NewCastle, PA, USA) according to the method reported elsewhere [20].

### Statistical analysis

Data are presented as mean±standard error of the mean. In *in vitro* study, data were analyzed by the one-way analysis of variance of SPSS 18.0 software, the potential differences among treatments were then detected using Duncan’s multiple range test. In *in vivo* study, data were analyzed by the mixed procedure of SPSS 18.0 software including the dietary treatment as the main fixed factor and the block as the random factor. For the data regarding growth performance and diarrhea rate, replicate was viewed as the experimental unit. For the other data, individual pig was treated as the experimental unit. The chi-square contingency test was employed to analyze the diarrhea rate. Significance was defined as p<0.05 and 0.05≤p<0.10 was considered to be a tendency toward significance.

## RESULTS

### *In vitro* experiments

#### Enzymological characteristics of Xyn, Afd, and FE

**Figure 1:**
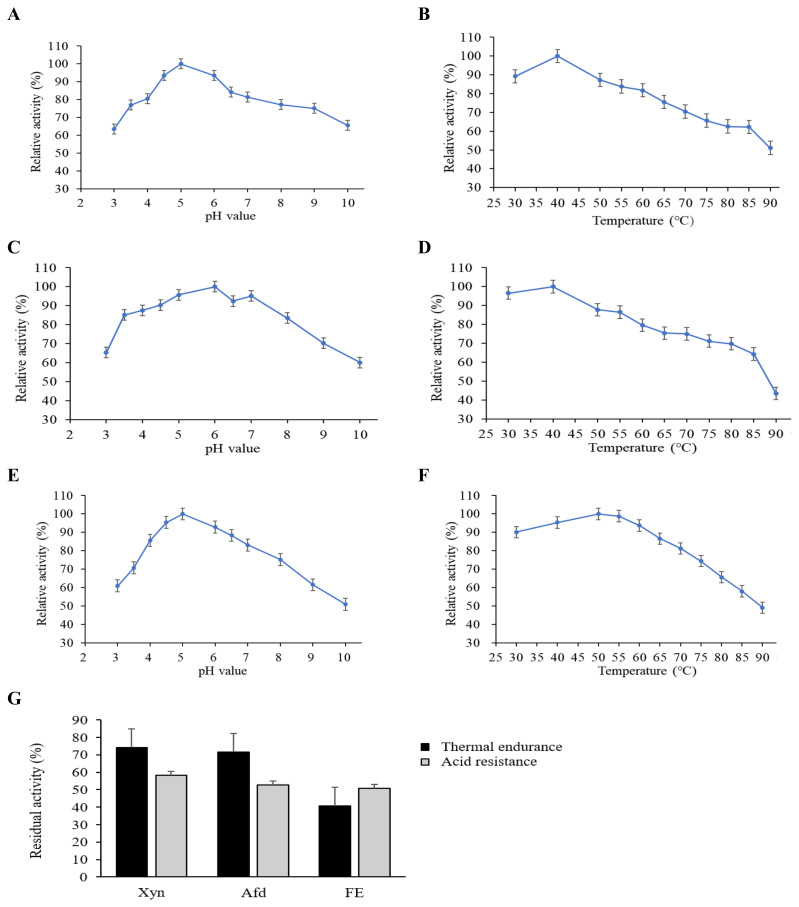
Enzymological characteristics of xylanase (Xyn), arabinfuranosidease (Afd), and feruloyl esterase (FE) (n = 3). The relative activities of Xyn (A, B), Afd (C, D), and FE (E, F) at different pH values and temperatures. The thermal endurance and acid resistance of Xyn, Afd, and FE (G). Thermal endurance and acid resistance were evaluated by determining the residual activities of these enzymes after incubation at 85°C for 3 min and at pH 3.0 for 30 min, respectively.

The activity of Xyn peaked at pH 5.0 and declined gently with the deviation of pH value from 5.0 (Figure 1A). More than 50% relative activity of Xyn was retained from 30°C to 90°C, with the value peaking at 40°C (Figure 1B). In terms of Afd, it had the highest activity at pH 6.0 and maintained more than 60% relative activity when pH value ranged from 3.0 to 9.0 (Figure 1C). The relative activity of Afd was kept above 60% from 30°C to 85°C and maximized at 40°C (Figure 1D). Regarding FE, over 60% relative activity was kept during pH 3.0 to 9.0 and the optimum pH was 5.0 (Figure 1E). Besides, it retained more than 60% relative activity during 30°C to 80°C, with 50°C being the optimal temperature (Figure 1F). Strikingly, both Xyn, Afd, and FE retained more than 50% relative activity when incubated at pH 3.0 for 30 min (Figure 1G). In addition, Xyn and Afd retained more than 70% relative activity while FE retained approximately 40% relative activity when underwent high temperature (85°C) treatment for 3 min.

#### Effect of Xyn combined with Afd on degradation of Abx in destarched wheat bran

As presented in Table 2, Xyn treatment alone increased (p<0.05) the RRS from destarched wheat bran. Combination of Xyn and Afd at a ratio ranging from 3,000:1 to 100:1 resulted in an increase (p<0.05) in the RRS from destarched wheat bran versus Xyn acting alone. However, the RRS from destarched wheat bran showed an irregular change in response to the increased doses of Afd.

#### Effect of Xyn combined with FE on degradation of Abx in destarched wheat bran

As shown in Table 3, combination of Xyn and FE at a ratio ranging from 3,000:1 to 100:1 induced an increase (p<0.05) in the RRS from destarched wheat bran relative to Xyn acting alone. The RRS from destarched wheat bran was not different among combined treatment groups, however, the numerical value of RRS peaked at the combination of Xyn and FE at a ratio of 500:1 (3 U/g Xyn + 0.006 U/g FE).

#### Synergy among Xyn, Afd, and FE on degradation of Abx in different brans

The yield of SF and IF of wheat bran, and of oat bran was 2.8% and 40.3%, and 7.4% and 8.7%, respectively (Table 4). While the extraction ratio of SF and IF from wheat bran, and from oat bran was 94.8% and 95.8%, 98. 7% and 95.2%, respectively. Combining Xyn with Afd increased (p<0.05) the RRS from all bran fibers when compared with Xyn acting alone (Table 5), comparatively, combining Xyn with FE increased (p<0.05) the RRS from SF and IF of oat bran rather than of wheat bran. Combination of Xyn and Afd elevated (p<0.05) the RRS from SF and IF of wheat bran versus combination of Xyn and FE, while combination of Xyn, Afd, and FE increased (p<0.05) the RRS from all bran fibers versus Xyn combining with Afd or FE. Regarding the synergy degree, it was found to be greater between Xyn and Afd than that between Xyn and FE. Notably, the synergy degree among Xyn, Afd, and FE was obviously greater than that between Xyn and Afd or FE.

#### Effect of combination of Xyn, Afd, and FE on in vitro digestion of bran-containing diet

As shown in Table 6, bran-containing diet treated with Xyn alone or combination of Xyn, Afd, and FE displayed a tendency (p<0.10) towards an increase in the *in vitro* digestibility of dry matter, crude protein, crude ash, and gross energy.

### *In vivo* experiment

#### Effect of combination of Xyn, Afd, and FE on growth performance of piglets fed bran-containing diet

As shown in Table 7, compared with Xyn acting alone, combination of Xyn, Afd, and FE was somewhat more beneficial resulting in increasing trends (p<0.10) of FBW, ADG, and G/F of piglets fed bran-containing diet. A reduction (p<0.05) was noted in the diarrhea rate of piglets in response to treatment with either Xyn alone or combination of Xyn, Afd, and FE, however, piglets treated with combination of Xyn, Afd, and FE had a lower (p<0.05) diarrhea rate than those treated with Xyn alone.

#### Effects of combination of Xyn, Afd, and FE on intestinal VFA profile and pH value of piglets fed bran-containing diet

As shown in Table 8 and 9, there were increases (p<0.05) in the concentrations of acetic acid and total VFA in both cecum and colon as well as butyric acid concentration in cecum of piglets treated with either Xyn alone or combination of Xyn, Afd, and FE, with higher (p<0.05) concentrations of these parameters induced by combination of Xyn, Afd, and FE relative to Xyn acting alone. Furthermore, an elevation (p<0.05) was recorded in cecal propionic acid concentration in piglets treated with combination of Xyn, Afd, and FE instead of Xyn acting alone, however, both combined enzyme treatment and Xyn treatment alone elevated (p<0.05) colonic propionic acid concentration of piglets. Regarding intestinal pH value, it was not found to be changed in response to Xyn treatment alone, nevertheless, combination of Xyn, Afd, and FE triggered a reduction (p<0.05) of cecal pH value together with a decreasing trend (p<0.10) of colonic pH value of piglets fed bran-containing diet.

## DISCUSSION

A study on the enzymological characteristics of Xyn, Afd, and FE can favor the understanding of their application potentials in animal diet. A previous study by Wang et al [13] indicated that over 70% of Xyn activity was kept between pH 3.0 and 10.0, while FE retained more than 80% of the activity at the pH range of 2.0 to 12.0, besides, both Xyn and FE were stable from 4°C to 40°C and drastically reduced its activities at temperature higher than 50°C. Similarly, this study revealed that both Xyn, Afd, and FE could maintain relatively high activities (more than 50% relative activity) within wide ranges of pH value (from 3.0 to 10.0) and temperature (from 30°C to 85°C). Moreover, Xyn, Afd, and FE particularly the first two had a relatively high thermal endurance and acid resistance, which could enable a feasibility of these enzymes applied in animal diets to withstand the hostile conditions during diet processing and gastrointestinal digestion.

The present result showed that Xyn treatment alone sharply increased the RRS from destarched wheat bran, hinting that Xyn exerted a leading role among combination of xylanolytic enzymes in degradation of Abx that subsequently released reducing sugars [5]. However, the existence of arabinose groups attached to Abx backbone could reduce the depolymerizing effect of Xyn on Abx, by hampering the recognition of cleavage sites within the backbone by Xyn [6,7]. But this steric hindrance may be alleviated by the usage of Afd that removes arabinose groups from side chains, thus enabling further action of Xyn on Abx [5–7]. In support of this view, we noted that combination of Xyn and Afd at various ratios (from 3,000:1 to 100:1) resulted in a more pronounced RRS from destarched wheat bran relative to Xyn acting alone, implying a cooperation between Xyn and Afd on degradation of Abx in destarched wheat bran [21]. Intriguingly, there was almost no elevation in the RRS with the increased proportion of Afd dose. Based on the overall consideration of efficacy and cost, the combination of Xyn and Afd at a ratio of 3,000:1 (3 U/g Xyn + 0.001 U/g Afd) seemed to be suitable. Thereby, this combination scheme of Xyn and Afd was used for further analysis.

The α-L-arabinofuranosyl residues linked in Abx backbone can further be substituted by ferulic acid ester [4,5], which acts as another essential side chain group of Abx to impede the depolymerization of Xyn, therefore retarding Abx degradation [6,10]. But this steric hindrance effect can be attenuated by the action of FE capable of hydrolyzing ferulic acid ester bonds attached to Abx side chains [7–9]. It has been found that the RRS from Abx-riched crop products under the synergy between Xyn and FE was higher than that under Xyn acting alone [13]. Similarly, this study demonstrated an advantage of combination of Xyn and FE at varying ratios (from 3,000:1 to 100:1) over Xyn alone in promoting the RRS from destarched wheat bran, which might emphasize a cooperation between Xyn and FE on degradation of Abx. Due to generation of the numerically highest RRS from destarched wheat bran, the combination of Xyn (3 U/g) and FE (0.006 U/g) at a ratio of 500:1 was selected for further analysis.

To validate the synergy among Xyn, Afd, and FE at selected doses (3, 0.001, and 0.006 U/g, respectively), SF and IF of wheat bran and oat bran were employed as different Abx sources. Remarkably, the present study revealed that combining Xyn with Afd had a superiority over Xyn alone to increase the RRS from all bran fibers, while combining Xyn with FE increased the RRS from SF and IF of oat bran instead of wheat bran. The difference between the degrading actions on oat bran fiber and wheat bran fiber might be related to their different level of ferulic acid ester that can be targeted by FE [22]. Although Abx contains a linear backbone substituted with various degrees of side chains including arabinose and ferulic acid [4,5], arabinose seems to be the main group linked as the side chain of Abx in most cereal brans [4]. It was thus deduced that Afd elicited a more important role than FE in removing the side chains of Abx, thus being more beneficial for the depolymerizing action of Xyn on Abx in brans. This might account for the current findings: i) combining Xyn with Afd caused a higher RRS from Abx sources (especially wheat bran fiber) relative to combination of Xyn and FE; ii) the synergy degree between Xyn and Afd was obviously greater than that between Xyn and FE. Strikingly, integration of Xyn, Afd, and FE increased the RRS from all bran fibers relative to combining Xyn with Afd or FE, which corresponded to the observed higher synergy degree among these three enzymes than that between Xyn and Afd or between Xyn and FE, highlighting that combining Xyn, Afd, and FE had an advantage over combining Xyn with Afd or FE in accelerating degradation of Abx in brans. This was most likely due to that cooperation of debranching enzymes (Afd and FE) was more efficient than their single action in eliminating the spatial obstacles (branch points) of Abx [5–7], thereby favoring the access of Xyn to Abx backbone and its depolymerization.

Treatment with Xyn has been confirmed to be effective in promoting digestion of dietary fiber in animals [23,24], nevertheless, few studies are available concerning the influence of Xyn in combination with debranching enzymes on nutrient digestibility of piglet diet. In the current study, combination of Xyn, Afd, and FE or Xyn alone tended to improve the *in vitro* digestibility of dry matter, crude protein, crude ash, and gross energy of piglet diet containing bran, with numerically higher digestibility of these nutrients induced by combined enzyme treatment relative to Xyn treatment alone. It seems that the efficient degradation of Abx under the observed synergy among Xyn, Afd, and FE potentially translated into a benefit for cell wall destruction of bran, which facilitated the access of digestive enzymes to the cell wall, intracellular or both constituents of bran [8–10], thus assisting with nutrient digestion of diet. Alternatively, the degradation of Abx in bran-containing diet under the action of combination of Xyn, Afd, and FE or of Xyn alone profited the reduction of digesta viscosity, allowing a sufficient contact between digesta and digestive enzymes [2,3]. This might be also partially responsible for the observed increase in *in vitro* nutrients = of diet exposed to enzyme treatments.

Previous studies evidenced a variable efficacy of Xyn treatment alone in enhancing pig growth performance [23,24]. We herein observed that combination of Xyn, Afd, and FE was more beneficial than Xyn alone to induce increasing trends of FBW, ADG, and G/F of piglets fed bran-containing diet. This was likely related to the observed increasing trend of nutrient digestibility of bran-containing diet treated with enzymes especially combination of Xyn, Afd, and FE. Alternatively, the potential production of xylooligosaccharides induced by Xyn or its combination with Afd and FE modulated gut microbiota [2], which possibly contributed to the improvements of piglet performance. Regarding the diarrhea rate of piglets, it was found to be reduced by the treatment with either Xyn alone or combination of Xyn, Afd, and FE, with a lower diarrhea rate found in combined treatment group versus Xyn alone group. It was probable that the elevated nutrient digestibility of diet induced by Xyn alone or combination of Xyn, Afd, and FE could reduce the nutrient residuals utilized by certain pathogens in hindgut, thereby decreasing diarrhea prevalence of piglets [25,26]. Besides, the more efficient degradation of dietary Abx under the synergy among Xyn, Afd, and FE might be more favorable to lower the viscosity of chyme, which was hypothesized to avoid overgrowth of intestinal harmful bacteria and subsequently reduce diarrhea rate of piglets [2,27].

Bacteria-produced VFA via fermentation of dietary fibers were characterized as a typical improver of gut health of pigs [20,28]. It was suggested that dietary Xyn treatment modulated fermentation pattern of gut microbiota and increased the concentrations of certain VFA in gut of pigs [23,24]. The present study indicated that combination of Xyn, Afd, and FE or Xyn alone could increase the concentrations of acetic acid and total VFA in both cecum and colon coupled with cecal butyric acid concentration of piglets fed bran-containing diet, with higher concentrations of these parameters observed in combined treatment group versus Xyn treatment alone. Moreover, an elevation was recorded in cecal and colonic propionic acid concentration of piglets due to the treatment with combination of Xyn, Afd, and FE. These results revealed a distinct advantage of combination of Xyn, Afd, and FE over Xyn alone in improving intestinal VFA profile, which might be due to that the efficient degradation of dietary Abx into oligoscaccharides under the synergy among Xyn, Afd, and FE could improve gut fermentation by several beneficial bacteria (namely the prebiotic effect) [2,24]. The elevated concentrations of certain VFA in gut following combined treatment with Xyn, Afd, and FE were postulated to translate into a lower pH value of gut. Herein, although treatment with Xyn alone had little influence on intestinal pH value, we indeed detected a reduction of cecal pH value together with a decreasing trend of colonic pH value in piglets treated with combination of Xyn, Afd, and FE. These results basically coincided to the simultaneous increases in cecal and colonic concentrations of VFA of piglets treated with combination of Xyn, Afd, and FE. It could be speculated that the increased concentrations of VFA coupled with the resulting decrease of pH value in gut due to combined treatment with Xyn, Afd, and FE were at least partially responsible for the observed reduction of diarrhea rate of piglets, since acid microenvironment in gut could resist pathogen invasion as well as boost gut health and growth performance of piglets [29,30].

## CONCLUSION

The present study evidenced relatively high thermal endurance and acid resistance of Xyn, Afd, and FE, thus being suitable to be applied in animal diet. Combination of these enzymes was superior to Xyn alone and its combination with Afd or FE in degrading Abx in brans. Dietary treatment with combination of Xyn, Afd, and FE was more beneficial than Xyn alone to ameliorate growth performance and intestinal VFA profile of piglets fed bran-containing diet. Overall, combination of Xyn, Afd, and FE had a superior efficacy compared with Xyn alone in improving utilization of cereal bran in piglet diet.

## Figures and Tables

**Table 1:** The composition and nutrient levels of basal diet (as-fed basis)

Item	Content (%)
Ingredients	
Corn	60
Soybean meal	15
Wheat bran	1.5
Soybean oil	0.45
Puffed soybean	6.9
Fish meal	4.5
Whey powder	7.75
Dicalcium phosphate	0.85
Limestone	1.1
Salt	0.3
Lysine	0.35
Threonine	0.15
Methionine	0.1
Choline	0.2
Silicon dioxide	0.3
Premix^1)^	0.55
Total	100
Nutrient levels	
Digestible energy (MJ/kg)	14.43
Metabolizable energy (MJ/kg)	13.90
Crude protein (%)	18.76
Standardized digestible lysine (%)	1.14
Standardized digestible methionine (%)	0.36
Calcium (%)	0.67
Available phosphorus (%)	0.33

1) Supplied per kilogram of diet: Cu, 9.9 mg (as copper sulphate); Zn, 142.5 mg (as zinc oxide); Fe, 242.5 mg (as ferrous sulphate); Mn, 68.7 mg (as manganous oxide); Se, 0.36 mg (as sodium selenite); I, 0.64 mg (as potassium iodine); vitamin A, 11,500 IU; vitamin D_3_, 3,250 IU; vitamin E, 91 mg; vitamin K_3_, 3 mg; thiamin, 8.6 mg; riboflavin, 10.9 mg; pyridoxine, 9.3 mg; cobalamin, 0.2 mg; niacin, 62.6 mg; pantothenic acid, 26.8 mg; folic acid, 1.9 mg.

**Table 2:** Effect of combination of xylanase (Xyn) and arabinfuranosidease (Afd) on the release of reducing sugar in destarched wheat bran (n = 5)

Treatments^1)^	Releasing of reducing sugar (mg/mL)
Control	0.4±0.1^c^
X	13.5±0.2^b^
X+A1 (3,000:1)	15.2±0.2^a^
X+A2 (1,000:1)	14.9±0.2^a^
X+A3 (500:1)	15.3±0.1^a^
X+A4 (100:1)	14.7±0.3^a^
p-value	<0.05

1) Control, free of enzymes; X, 3 U/g Xyn; X+A1, 3 U/g Xyn + 0.001 U/g Afd; X+A2, 3 U/g Xyn + 0.003 U/g Afd; X+A3, 3 U/g Xyn + 0.006 U/g Afd; X+A4, 3 U/g Xyn + 0.03 U/g Afd.

a–c Values with different superscripts within the same column differ significantly (p<0.05).

**Table 3:** Effect of combination of xylanase (Xyn) and feruloyl esterase (FE) on the release of reducing sugar in destarched wheat bran (n = 5)

Treatments^1)^	Releasing of reducing sugar (mg/mL)
Control	0.4±0.1^c^
X	13.2±0.1^b^
X+F1 (3,000:1)	13.9±0.1^a^
X+F2 (1,000:1)	14.1±0.1^a^
X+F3 (500:1)	14.5±0.1^a^
X+F4 (100:1)	14.4±0.1^a^
p-value	<0.05

1) Control, free of enzymes; X, 3 U/g Xyn; X+F1, 3 U/g Xyn + 0.001 U/g FE; X+F2, 3 U/g Xyn + 0.003 U/g FE; X+F3, 3 U/g Xyn + 0.006 U/g FE; X+F4, 3 U/g Xyn + 0.03 U/g FE.

a–c Values with different superscripts within the same column differ significantly (p<0.05).

**Table 4:** Extraction of fibers from wheat bran and oat bran

Items	Wheat bran		Oat bran	
	
	Yield^1)^ (%)	Extraction ratio^2)^ (%)	Yield (%)	Extraction ratio (%)
Soluble fiber	2.8	94.8	7.4	98.7
Insoluble fiber	40.3	95.8	8.7	95.2

1) Yield (%) means the percentage of the weight of extracted fiber to the weight of bran.

2) Extraction ratio (%) means the percentage of the weight of extracted fiber to the weight of fiber included in bran.

**Table 5:** Synergy among xylanase (Xyn), arabinfuranosidease (Afd), and feruloyl esterase (FE) on degradation of arabinoxylan from different sources^1)^ (n = 5)

Treatments^2)^	WB-SF		WB-IF		OB-SF		OB-IF	
			
	Reducing sugar (mg/mL)	Degree of synergy^3)^	Reducing sugar (mg/mL)	Degree of synergy	Reducing sugar (mg/mL)	Degree of synergy	Reducing sugar (mg/mL)	Degree of synergy
X	24.1±0.3^c^	1.0	6.9±0.2^c^	1.0	21.4±0.1^c^	1.0	8.0±0.1^c^	1.0
X+A	32.3±0.2^b^	1.3	11.4±0.2^b^	1.7	29.1±0.3^b^	1.4	10.6±0.2^b^	1.3
X+F	26.7±0.2^c^	1.1	7.3±0.1^c^	1.1	25.5±0.2^b^	1.2	9.0±0.1^b^	1.1
X+A+F	42.7±0.2^a^	1.8	13.0±0.1^a^	1.9	37.2±0.4^a^	1.7	13.3±0.1^a^	1.7
p-value	0.05	-	0.05	-	0.05	-	0.05	-

1) WB-SF, soluble fiber of wheat bran; WB-IF, insoluble fiber of wheat bran; OB-SF, soluble fiber of oat bran; OB-IF, insoluble fiber of oat bran.

2) X, treatment with 3 U/g Xyn; X+A, treatment with combination of 3 U/g Xyn and 0.001 U/g Afd; X+F, treatment with combination of 3 U/g Xyn and 0.006 U/g FE; X+A+F, treatment with combination of 3 U/g Xyn, 0.001 U/g Afd and 0.006 U/g FE.

3) The value of synergy degree between multiple enzymes was calculated as the ratio of the sum of the activities (generation amount of hydrolysate) of all enzymes to the activity of one of these enzymes.

a–c Values with different superscripts within the same column differ significantly (p<0.05).

**Table 6:** Effect of combination of xylanase (Xyn), arabinfuranosidease (Afd), and feruloyl esterase (FE) on *in vitro* digestibility of nutrients of bran-containing diet (n = 5)

Treatments^1)^	Digestibility (%)			

	Dry matter	Crude protein	Crude ash	Gross energy
Control	71.1±0.1	65.8±0.3	51.3±0.8	64.0±0.2
X	74.1±0.3	68.9±0.1	54. 5±0.8	67.1±0.2
X+A+F	74.6±0.2	69.5±0.2	55.0±0.6	67.8±0.2
p-value	0.05≤p<0.10	0.05≤p<0.10	0.05≤p<0.10	0.05≤p<0.10

1) Control, free of enzymes; X, treatment with 3 U/g Xyn; X+A+F, treatment with combination of 3 U/g Xyn, 0.001 U/g Afd and 0.006 U/g FE.

**Table 7:** Effect of combination of xylanase (Xyn), arabinfuranosidease (Afd), and feruloyl esterase (FE) on growth performance^1)^ of piglets received bran-containing diet (n = 6)

Treatments^2)^	IBW (kg)	FBW (kg)	ADG (g)	ADFI (g)	G/F	DR (%)
Control	9.70±0.05	17.1±0.2	351±7	608±7	0.578±0.003	0.87±0.11^a^
X	9.71±0.04	17.6±0.3	378±6	616±7	0.613±0.006	0.55±0.07^b^
X+A+F	9.69±0.05	17.9±0.2	391±7	626±5	0.625±0.013	0.17±0.08^c^
p-value	p>0.10	0.05≤p<0.10	0.05≤p<0.10	p>0.10	0.05≤p<0.10	p<0.05

1) IBW, initial body weight; FBW, final body weight; ADG, average daily gain; ADFI, average daily feed intake; G/F, gain to feed ratio; DR, diarrhea rate.

2) Control, free of enzymes; X, treatment with 1,600 U/kg Xyn; X+A+F, treatment with combination of 1,600 U/kg Xyn, 0.8 U/kg Afd and 4 U/kg FE.

a–c Values with different superscripts within the same column differ significantly (p<0.05).

**Table 8:** Effects of combination of xylanase (Xyn), arabinfuranosidease (Afd), and feruloyl esterase (FE) on volatile fatty acids (VFA) concentrations and pH value in cecal digesta of piglets fed bran-containing diet (n = 6)

Treatments^1)^	Acetic acid (mg/g)	Propionic acid (mg/g)	Butyric acid (mg/kg)	Total VFA (mg/g)	pH value
Control	2.86±0.33^a^	0.82±0.21^a^	32.14±8.52^a^	3.92±0.22^a^	6.17±0.41^a^
X	3.41±0.28^b^	1.07±0.13^ab^	41.56±9.63^b^	4.96±0.14^b^	5.98±0.33^a^
X+A+F	4.51±0.46^c^	1.29±0.20^b^	78.94±9.85^c^	6.39±0.77^c^	5.44±0.19^b^
p-value	p<0.05	p<0.05	p<0.05	p<0.05	p<0.05

1) Control, free of enzymes; X, treatment with 1,600 U/kg Xyn; X+A+F, treatment with combination of 1,600 U/kg Xyn, 0.8 U/kg Afd and 4 U/kg FE.

a–c Values with different superscripts within the same column differ significantly (p<0.05).

**Table 9:** Effects of combination of xylanase (Xyn), arabinfuranosidease (Afd), and feruloyl esterase (FE) on volatile fatty acids (VFA) concentrations and pH value in colonic digesta of piglets fed bran-containing diet (n = 6)

Treatments^1)^	Acetic acid (mg/g)	Propionic acid (mg/g)	Butyric acid (mg/kg)	Total VFA (mg/g)	pH value
Control	2.01±0.39^a^	0.66±0.44^a^	66.97±9.36	2.91±0.50^a^	6.46±0.11
X	3.69±0.47^b^	0.96±0.36^b^	68.28±8.99	5.00±0.46^b^	6.27±0.19
X+A+F	4.88±0.58^c^	1.01±0.30^b^	67.40±8.77	6.02±0.61^c^	6.03±0.30
p-value	p<0.05	p<0.05	p>0.10	p<0.05	0.05≤p<0.10

1) Control, free of enzymes; X, treatment with 1,600 U/kg Xyn; X+A+F, treatment with combination of 1,600 U/kg Xyn, 0.8 U/kg Afd and 4 U/kg FE.

a–c Values with different superscripts within the same column differ significantly (p<0.05).
